# Male subfertility and the risk of major birth defects in children born after in vitro fertilization and intracytoplasmic sperm injection: a retrospective cohort study

**DOI:** 10.1186/s12884-019-2322-7

**Published:** 2019-06-03

**Authors:** Seung Chik Jwa, Junna Jwa, Akira Kuwahara, Minoru Irahara, Osamu Ishihara, Hidekazu Saito

**Affiliations:** 1 Division of Reproductive Medicine, Center of Maternal–Fetal, Neonatal and Reproductive Medicine, National Center for Child Health and Development, 2-10-1, Okura, Setagaya-ku, Tokyo, 157-8535 Japan; 2 Department of Obstetrics and Gynecology, Saitama Medical University, 38 Morohongo, Moroyama, Saitama, 350-0495 Japan; 3 Department of Obstetrics and Gynecology, Graduate School of Biomedical Sciences, University of Tokushima, 3-18-5, Kuramoto-chou, Tokushima-shi, Tokushima, 770-8503 Japan

**Keywords:** Assisted reproductive technology, Birth defect, Intracytoplasmic sperm injection, In vitro fertilization, Male infertility

## Abstract

**Background:**

Children born after intracytoplasmic sperm injection (ICSI) are at increased risk of specific major birth defects compared with children born after in vitro fertilization (IVF). However, whether this risk is due to the treatment itself (i.e., IVF or ICSI) or underlying male subfertility is unknown. This study investigated the associations between male subfertility and the risk of major birth defects in children born after IVF and ICSI.

**Methods:**

We conducted a retrospective cohort study using data from the Japanese assisted reproductive technology registry between 2007 and 2014. Fresh embryo transfer cycles registered from 2007 to 2014 that resulted in singleton live births, still births, or selective terminations were included (*n* = 59,971). Major birth defects were defined by the US Centers for Disease Control and Prevention guidelines, excluding chromosomal abnormalities. Odds ratios (ORs) and 95% confidence intervals (CIs) were calculated using generalized estimating equations adjusting for potential confounders.

**Results:**

Major birth defects were reported in 626/59,971 (1.04%) cases. Among IVF cycles, male subfertility was associated with significantly greater risks of hypospadias (3/3163 [0.09%] vs 4/28,671 [0.01%], adjusted OR = 6.85, 95% CI 2.05–22.9, *P* = 0.002) and atrial septal defects (4/3163 [0.13%] vs 9/28,671 [0.03%], adjusted OR = 3.98, 95% CI 1.12–14.1, *P* = 0.03) compared with fertile men. Subgroup analysis using sperm parameters showed that oligozoospermia (i.e., sperm concentrations < 15 million/mL) was significantly associated with a greater risk of ventricular septal defects compared with normal sperm concentrations in IVF pregnancies (5/868 [0.58%] vs 60/28,090 [0.21%], adjusted OR = 2.68, 95% CI 1.15–6.27, *P* = 0.02), and severe oligozoospermia (i.e., sperm concentrations < 5 million/mL) was significantly associated with an increased risk of hypospadias compared with normal sperm concentrations in ICSI pregnancies (5/3136 [0.16%] vs 5/16,865 [0.03%], adjusted OR = 3.88, 95% CI 1.14–13.2, *P* = 0.03).

**Conclusions:**

The results of this exploratory study suggest that underlying male subfertility may play a role in the risk of major birth defects related to ICSI and IVF. Further research, including systematic reviews adjusting for confounders, is required to confirm the associations between male subfertility and major cardiac and urogenital birth defects.

**Electronic supplementary material:**

The online version of this article (10.1186/s12884-019-2322-7) contains supplementary material, which is available to authorized users.

## Background

The first neonate born as a result of in vitro fertilization (IVF) was born in the United Kingdom in 1978 [1]. Since this time, assisted reproductive technology (ART), including IVF and intracytoplasmic sperm injection (ICSI), has been widely used in infertility treatments worldwide, with more than 1 million newborns reportedly born as a result of IVF or ICSI between 2008 and 2010 [2]. The trend is similar in Japan, with 51,001 neonates born following ART in 2015, accounting for almost 1 in 19.7 births [3].

Despite the dramatic increase in ART-related pregnancies, the safety of these techniques continues to be a matter of concern. Children born after IVF/ICSI are at increased risk of major birth defects compared with children born after natural conception [4]. However, previous studies have demonstrated conflicting results regarding the associations between ICSI and major congenital anomalies. Two meta-analyses that investigated the incidence of birth defects in children born after ICSI and IVF both failed to find any significant difference between the two techniques [5, 6], while a more recent population-based cohort study from Australia reported a significantly greater risk of birth defects after ICSI than after IVF (odds ratio [OR] = 1.47, 95% confidence interval [95% CI] 1.15–1.89) [7]. Furthermore, recent studies showed that children born after ICSI had significantly increased risks of urogenital anomalies (OR = 1.27, 95% CI 1.02–1.59) [8] and specific major cardiac birth defects [9] compared with children born after IVF. However, caution should be exercised in interpreting these results given that ICSI has recently been used for various indications other than male subfertility, and whether the increased risk of specific major birth defects in children born after ICSI is due to the technique itself or to any underlying male subfertility is unknown [10]. To the best of our knowledge, no studies have attempted to distinguish between the effects of treatment (i.e., IVF/ICSI) and that of underlying male subfertility on the risk of major birth defects.

We therefore conducted an exploratory analysis to evaluate the associations between male subfertility and the risk of major birth defects in children born following IVF and ICSI, using a nationally representative ART sample from Japan.

## Methods

### Data source and study sample

We conducted a registry-based, retrospective cohort study using a Japanese national ART registry database assembled by the Japan Society of Obstetrics and Gynecology (JSOG). All ART clinics and hospitals are required to contribute to this database, which has previously been reported in detail [11]. The data comprised all cycle-specific information, including infertility diagnosis, IVF or ICSI, and pregnancy and obstetric outcomes. JSOG requires all participating clinics or hospitals with delivery facilities to report delivery outcomes to the ART registry. ART clinics without delivery facilities usually receive delivery reports from referral delivery hospitals or clinics and then report these to the registry. JSOG strongly recommends that ART clinics or hospitals that cannot obtain such delivery reports contact the mothers directly to obtain self-reported obstetrical outcomes. JSOG prohibits the use of donor gametes or embryos, and all embryos transferred were therefore autologous. Similarly, preimplantation genetic testing (PGT) for chromosomal aneuploidy is not allowed in Japan, and PGT for monogenic or structural rearrangements is only allowed after approval by both the institutional review board at each facility and the JSOG board of ethics. A total of 671 cycles of oocyte retrieval involving PGT for monogenic or structural rearrangements were reported between September 2004 and September 2012 in Japan, resulting in 65 singleton births [12].

Fertilization-related information, such as IVF or ICSI status, was only available for fresh cycles. We therefore included all singleton live births and stillbirths after 22 weeks of gestation, or those with a birth weight > 500 g in cases of unknown gestational age, after fresh embryo transfer cycles. We also included pregnancies that were terminated because of birth defects. Overall, 69,346 cycles were eligible for analysis in this study (68,832 cycles for live births, 284 for stillbirths, and 230 for selectively terminated cases). Among these, we excluded cycles using previously frozen oocytes (*n* = 24), gamete intra-Fallopian transfers (*n* = 7), split-ICSI (*n* = 6493) or ICSI using non-ejaculated sperm (*n* = 1298), unknown fertilization methods (*n* = 262), and other embryo stages at transfer (*n* = 1291). A total of 59,971 cycles (59,527 live births, 248 stillbirths, and 196 selectively terminated pregnancies) were included in the final analysis.

### Ethical approval

This study was approved by the Institutional Review Board of the National Center for Child Health and Development (Institutional Review Board Approval no. 34), Saitama Medical University (Institutional Review Board Approval no. 873), and the JSOG Ethics Committee (Approval no. IM007). After receipt of approval, JSOG provided the required data without any personal identifying information. We analyzed data from 2007 to 2014 because JSOG implemented mandatory online reporting for all institutions from 2007, and the most recent data available at the time of this study were from 2014.

### Definition of major birth defects

We included major birth defects that were identified before the end of the neonatal period (within 28 days). We defined major birth defects according to the US Centers for Disease Control and Prevention (CDC) guidelines [13]. These birth defects were classified based on blinded review of abstraction forms by a medical doctor who had completed residencies and was board-certified in obstetrics and pediatrics (J.J.). Fetuses and infants with minor anomalies as defined by the CDC, with complications caused by prematurity, and with unspecified conditions or suspected anomalies without definite diagnoses were defined as having no major anomalies. Classification of major birth defects according to organ systems does not suitably express the overall panorama of major birth defects of different natures. We therefore investigated the most common specific major birth defects that occurred in sufficient numbers to examine each organ system. These birth defects included ventricular septal defect, atrial septal defect, tetralogy of Fallot, omphalocele, gastroschisis, diaphragmatic hernia, polydactyly, syndactyly, hypospadias, alimentary atresia (esophageal, small intestine, and rectal and large intestinal atresia), anencephaly (including acrania), spina bifida, and cleft lip with and without cleft palate. To create more uniform case categories, cases with chromosomal abnormalities (*n* = 279) were excluded from the major birth defects, including 271 (97.1%) cases of chromosomal aneuploidy, four cases of deletion, two cases of inversion, one case of unbalanced translocation, and one case of additional chromosomal material. Among the eight cases of chromosomal abnormalities other than chromosomal aneuploidy, there were only two cases of major congenital anomalies. Conditions that were not defined by the CDC were discussed by a board-certified obstetrician and pediatrician (J.J) and a board-certified obstetrician and clinical geneticist (S.C.J.), blinded to the methods of conception. These conditions were classified as major birth defects based on the presence of structural abnormalities causing functional impairment and requiring surgical correction.

### Other variables

Infertility diagnosis was classified in the registry as tubal factor, male subfertility, endometriosis, antisperm antibody, unexplained, or other, with multiple answers allowed. Male subfertility included abnormal results of semen analysis or sexual or ejaculation dysfunction, and was diagnosed in each institution. Patients who failed to meet any of the specific infertility diagnoses including male subfertility were defined as having unexplained infertility. Sperm parameters, including total sperm motility and sperm concentrations used for fertilization, were available for 52,968 cycles (88.3% of the total sample). Total sperm motility (%) was recorded in the database to the nearest integer, and sperm concentrations (× 10^6^/mL) were calculated to two decimal places. We defined oligozoospermia as a sperm concentration < 15 × 10^6^/mL, asthenozoospermia as total sperm motility < 40%, and oligoasthenozoospermia as both of the above, according to the World Health Organization classification of subfertility [14].

### Statistical analysis

We first compared baseline characteristics between fertile and subfertile men in IVF/ICSI cycles using χ^2^ or Student’s *t*-tests. Second, we assessed the effect of male subfertility on major birth defects by comparing the prevalence of major birth defects in fertile and subfertile men within IVF or ICSI cycles. We calculated ORs and 95% CIs using generalized estimating equations with robust variance estimation, adjusting for the effect of clustering of births within clinics or hospitals. Our potential confounders included for adjusted analysis were maternal age (categorized into 5-year age groups), calendar year, embryo stage at transfer, and fetal sex. Analysis was restricted to male infants for the outcome of hypospadias. Third, the effect of treatment (i.e., IVF/ICSI) was assessed by comparing the prevalence of major birth defects between IVF and ICSI among fertile and subfertile men, and ORs of ICSI compared with IVF were calculated for major birth defects. Finally, we performed subgroup analyses of cycles for which sperm parameters were available by calculating ORs of different thresholds of sperm concentrations/motility for major birth defects with ICSI and IVF. These thresholds were defined as oligozoospermia (< 15 × 10^6^/mL) and severe oligozoospermia (< 5 × 10^6^/mL), and sperm motility was defined as asthenozoospermia (total sperm motility < 40%) and severe asthenozoospermia (total sperm motility < 25%). Because the number of cases of oligozoospermia was small in IVF cycles (*n* = 868), we did not stratify the category further into severe oligozoospermia. We also investigated the risk of major birth defects in ICSI among cycles with normal semen concentrations and motility. Adjustments for multiple comparisons were not applied because of the exploratory nature of this study. All analyses were performed using the STATA SE statistical package, version 12.1 (Stata, College Station, TX, USA). A two-tailed value of *P* < 0.05 was considered statistically significant.

## Results

The baseline characteristics of the sample cycles stratified by IVF and ICSI with or without male subfertility are shown in Table 1. There were 28,671 cycles of IVF from fertile men and 3163 cycles from subfertile men, and 13,777 cycles of ICSI from fertile men and 14,360 cycles from subfertile men. The mean maternal age was highest in ICSI cycles from fertile men. Infertility diagnoses of tubal factor and endometriosis were more frequent in IVF and ICSI from fertile compared with subfertile men.

**Table 1:** Characteristics of the sample population stratified by IVF and ICSI with or without male subfertility (2007–2014, *n* = 59,971 cycles)

Characteristics	IVF			ICSI		
	Fertile men (n = 28,671)	Subfertile men (*n* = 3163)	*P* value^e^	Fertile men (*n* = 13,777)	Subfertile men (*n* = 14,360)	*P* value^e^
Maternal age (y)	35.1 ± 3.8	35.2 ± 3.8	0.43	35.8 ± 3.8	34.8 ± 3.9	<.001
< 30	2275 (7.9)	240 (7.6)	0.84	783 (5.7)	1395 (9.7)	<.001
30–34	9801 (34.2)	1076 (34.0)		3995 (29.0)	4943 (34.4)	
35–39	13,091 (45.7)	1466 (46.4)		6584 (47.8)	6411 (44.6)	
≥ 40	3504 (12.2)	381 (12.1)		2415 (17.5)	1611 (11.2)	
Infertility diagnosis^a^						
Tubal factor	6761 (23.6)	432 (13.7)	<.001	2479 (18.0)	1143 (8.0)	<.001
Endometriosis	2756 (9.6)	244 (7.7)	0.001	1188 (8.6)	660 (4.6)	<.001
Antisperm antibody	232 (0.81)	15 (0.47)	0.04	170 (1.23)	38 (0.26)	<.001
Other	3507 (12.2)	513 (16.2)	<.001	2513 (18.2)	1700 (11.8)	<.001
Unexplained	16,828 (58.7)	–	–	8150 (59.2)	–	–
Quality of semen for fertilization	(n = 26,056)	(*n* = 2902)		(n = 12,214)	(*n* = 11,796)	
Sperm concentration, (×10^6^/mL)	101.0 ± 462.6	71.3 ± 238.6	<.001	66.9 ± 244.1	32.7 ± 80.9	<.001
Total sperm motility, (%)	58.4 ± 16.1	49.7 ± 18.5	<.001	47.9 ± 19.8	34.7 ± 20.9	<.001
Normal sperm	22,418 (86.0)	1901 (65.5)	<.001	7574 (62.0)	2946 (25.0)	<.001
Oligozoospermia^b^	447 (1.7)	169 (5.8)		761 (6.2)	1772 (15.0)	
Asthenozoospermia^b^	3050 (11.7)	721 (24.8)		2872 (23.5)	3473 (29.4)	
Oligoasthenozoospermia^b^	141 (0.54)	111 (3.8)		1007 (8.2)	3605 (30.6)	
Embryo stage at transfer						
Early cleavage	20,232 (70.6)	2206 (69.7)	0.34	9998 (72.6)	9792 (68.2)	<.001
Blastocyst	8439 (29.4)	957 (30.3)		3779 (27.4)	4568 (31.8)	
Number of embryos transferred^c^						
One	21,798 (76.0)	2377 (75.2)	0.55	10,160 (73.8)	10,330 (71.9)	0.002
Two	6314 (22.0)	723 (22.9)		3359 (24.4)	3767 (26.2)	
Three or more	559 (2.0)	63 (2.0)		258 (1.9)	263 (1.8)	
Year^d^						
2007	3154 (49.1)	335 (5.2)	0.02	1232 (19.2)	1707 (26.6)	<.001
2008	3299 (50.1)	345 (5.2)		1282 (19.5)	1660 (25.2)	
2009	3687 (48.8)	438 (5.8)		1520 (20.1)	1914 (25.3)	
2010	3537 (47.5)	390 (5.2)		1569 (21.1)	1951 (26.2)	
2011	3494 (46.4)	415 (5.5)		1701 (22.6)	1920 (25.5)	
2012	3782 (47.9)	355 (4.5)		2010 (25.4)	1753 (22.2)	
2013	3793 (46.7)	415 (5.1)		2180 (26.8)	1741 (21.4)	
2014	3925 (46.8)	470 (5.6)		2283 (27.2)	1714 (20.4)	

Data are mean ± standard deviation or n (%) unless otherwise specified

*IVF* in vitro fertilization, *ICSI* intracytoplasmic sperm injection

^a^Multiple diagnoses were allowed. Patients who did not have any specific infertility diagnosis among subfertile men had an infertility diagnosis of male subfertility alone. Patients who met neither of any infertility diagnosis including male subfertility were defined as unexplained infertility

^b^Oligozoospermia was defined by sperm concentrations < 15 × 10^6^ spermatozoa/mL, asthenozoospermia as total sperm motility < 40%, and oligoasthenozoospermia as both

^c^Only singleton births were included in the table

^d^Percentages are shown in a row for the purpose of comparison

^e^*P* values were assessed by using the χ^2^ test or Student’s t-test between fertile and subfertile men among IVF/ICSI cycles

The prevalence and ORs of subfertile men compared with fertile men for major birth defects in IVF cycles are shown in Table 2. Major birth defects were observed in 273/28,671 (0.95%) cycles from fertile men and 40/3163 (1.26%) from subfertile men, with no significant difference (*P* = 0.09). However, male subfertility was associated with significantly higher ORs for atrial septal defects (4/3163 [0.13%] vs 9/28,671 [0.03%], adjusted OR = 3.98, 95% CI 1.12–14.1, *P* = 0.03) and hypospadias (3/3163 [0.09%] vs 4/28,671 [0.01%], adjusted OR = 6.85, 95% CI 2.05–22.9, *P* = 0.002) compared with fertile men. .

**Table 2:** Prevalence of major birth defect, ORs and 95% CIs of subfertile men compared with fertile men for major birth defects within IVF (n = 31,834 cycles)

Type of major birth defect	IVF			
	n (%)		Crude OR (95% CI)	Adjusted OR (95% CI)^c^
	Fertile men (n = 28,671)	Subfertile men (*n* = 3163)		
Any major anomaly	273 (0.95)	40 (1.26)	1.33 (0.82 to 2.15)	1.35 (0.83 to 2.20)
Cardiovascular				
Ventricular septal defect	57 (0.20)	11 (0.35)	1.75 (0.87 to 3.51)	1.73 (0.86 to 3.51)
Atrial septal defect	9 (0.03)	4 (0.13)	4.03 (1.11 to 14.6)	3.98 (1.12 to 14.1)
Tetralogy of Fallot	11 (0.04)	2 (0.06)	1.65 (0.38 to 7.09)	1.64 (0.38 to 7.00)
Musculoskeletal				
Omphalocele	1 (0.00)	1 (0.03)	9.07 (0.56 to 147.9)	–
Gastroschisis	2 (0.01)	0 (0)	–	–
Diaphragmatic hernia	4 (0.01)	1 (0.03)	2.27 (0.23 to 22.1)	2.44 (0.24 to 24.9)
Polydactyly	25 (0.09)	2 (0.06)	0.72 (0.17 to 3.07)	0.72 (0.17 to 3.10)
Syndactyly	9 (0.03)	0 (0.0)	–	–
Urogenital				
Hypospadias^a^	4 (0.01)	3 (0.09)	6.77 (1.99 to 23.1)	6.85 (2.05 to 22.9)
Gastrointestinal				
Alimentary atresia^b^	15 (0.05)	1 (0.03)	0.60 (0.08 to 4.73)	0.61 (0.08 to 4.80)
Esophageal atresia	6 (0.02)	0 (0)	–	–
Atresia of small intestine	2 (0.01)	0 (0)	–	–
Rectal and large intestinal atresia	7 (0.02)	1 (0.03)	1.30 (0.15 to 11.2)	1.34 (0.16 to 11.5)
Central nervous system				
Anencephaly	23 (0.08)	0 (0)	–	–
Spina bifida	8 (0.03)	0 (0)	–	–
Orofacial				
Cleft lip with and without cleft palate	18 (0.06)	2 (0.09)	1.51 (0.46 to 4.99)	1.56 (0.48 to 5.07)

*OR* odds ratio, *IVF* in vitro fertilization

^a^Analysis was restricted within male infants

^b^Alimentary atresia is a composite outcomes of esophageal atresia, atresia of small intestine and rectal and large intestinal atresia

^c^adjusted for maternal age, calendar year, embryo stage at transfer, and fetal sex

The prevalence of and ORs of subfertile men compared with fertile men for major birth defects in ICSI cycles are shown in Table 3. Major birth defects were observed in 156/13,777 (1.13%) cycles from fertile men and 157/14,360 (1.09%) from subfertile men, with no significant difference (*P* = 0.76). Male subfertility was associated with a significantly higher OR for hypospadias (10/14,360 [0.07%] vs 3/13,777 [0.02%], crude OR = 2.89, 95% CI 1.002–8.33, *P* = 0.049) compared with fertile men, but this relationship became non-significant after adjusting for potential confounders (adjusted OR = 2.65, 95% CI 0.90–7.85, *P* = 0.08).

**Table 3:** Prevalence of major birth defect, ORs and 95% CIs of subfertile men compared with fertile men for major birth defects within ICSI (n = 28,137 cycles)

Type of major birth defect	ICSI			
	n (%)		Crude OR (95% CI)	Adjusted OR (95% CI)^c^
	Fertile men (n = 13,777)	Subfertile men (n = 14,360)		
Any major anomaly	156 (1.13)	157 (1.1)	0.97 (0.68 to 1.38)	0.95 (0.67 to 1.35)
Cardiovascular				
Ventricular septal defect	40 (0.29)	43 (0.30)	1.03 (0.58 to 1.83)	1.12 (0.65 to 1.91)
Atrial septal defect	8 (0.06)	7 (0.05)	0.84 (0.36 to 1.94)	0.75 (0.33 to 1.70)
Tetralogy of Fallot	2 (0.01)	3 (0.02)	1.44 (0.28 to 7.35)	1.78 (0.32 to 9.92)
Musculoskeletal				
Omphalocele	1 (0.01)	2 (0.01)	1.92 (0.17 to 21.6)	1.36 (0.13 to 13.9)
Gastroschisis	0 (0)	0 (0)	–	–
Diaphragmatic hernia	6 (0.04)	3 (0.02)	0.48 (0.13 to 1.83)	0.49 (0.12 to 2.08)
Polydactyly	11 (0.08)	5 (0.03)	0.44 (0.16 to 1.19)	0.37 (0.14 to 1.01)
Syndactyly	3 (0.02)	3 (0.02)	0.96 (0.21 to 4.40)	0.87 (0.14 to 5.26)
Urogenital				
Hypospadias^a^	3 (0.02)	10 (0.07)	2.89 (1.002 to 8.33)	2.65 (0.90 to 7.85)
Gastrointestinal				
Alimentary atresia^b^	13 (0.09)	15 (0.10)	1.11 (0.52 to 2.35)	0.96 (0.46 to 1.98)
Esophageal atresia	4 (0.03)	4 (0.03)	0.96 (0.24 to 3.90)	0.66 (0.17 to 2.50)
Atresia of small intestine	2 (0.01)	2 (0.01)	0.96 (0.11 to 8.08)	0.97 (0.14 to 6.82)
Rectal and large intestinal atresia	8 (0.06)	9 (0.06)	1.08 (0.41 to 2.86)	0.99 (0.39 to 2.53)
Central nervous system				
Anencephaly	5 (0.04)	5 (0.03)	0.96 (0.28 to 3.24)	0.85 (0.22 to 3.27)
Spina bifida	7 (0.05)	2 (0.01)	0.27 (0.06 to 1.31)	0.26 (0.06 to 1.11)
Orofacial				
Cleft lip with and without cleft palate	12 (0.09)	11 (0.08)	0.88 (0.37 to 2.11)	0.95 (0.40 to 2.25)

*OR* odds ratio, *CI* confidence interval, *ICSI* intracytoplasmic sperm injection

^a^Analysis was restricted within male infants

^b^Alimentary atresia is a composite outcomes of esophageal atresia, atresia of small intestine and rectal and large intestinal atresia

^c^adjusted for maternal age, calendar year, embryo stage at transfer, and fetal sex

The ORs of ICSI compared with IVF for major birth defects among fertile men and subfertile men are shown in Tables 4 and 5, respectively. ICSI was associated with a significantly greater risk of diaphragmatic hernia compared with IVF among fertile men (6/13,777 [0.04%] vs 4/28,671 [0.01%], adjusted OR = 3.53, 95% CI 1.15–10.9, *P* = 0.03), but there were no significantly increased ORs of ICSI for other major birth defects. There were no significant associations between ICSI and specific major birth defects among subfertile men (Table 5).

**Table 4:** ORs and 95% CIs of ICSI compared with IVF for major birth defects among fertile men (*n* = 42,448 cycles)

Type of major birth defect	Crude OR (95% CI)		Adjusted OR (95% CI)^c^	
	IVF	ICSI	IVF	ICSI
Any major anomaly	Ref.	1.19 (0.94 to 1.50)	Ref.	1.21 (0.96 to 1.52)
Cardiovascular				
Ventricular septal defect	Ref.	1.46 (0.94 to 2.28)	Ref.	1.38 (0.90 to 2.10)
Atrial septal defect	Ref.	1.85 (0.55 to 6.27)	Ref.	1.81 (0.50 to 6.63)
Tetralogy of Fallot	Ref.	0.38 (0.10 to 1.49)	Ref.	0.38 (0.10 to 1.47)
Musculoskeletal				
Omphalocele	Ref.	2.08 (0.13 to 33.7)	Ref.	2.14 (0.12 to 36.9)
Gastroschisis	Ref.	–	Ref.	–
Diaphragmatic hernia	Ref.	3.12 (1.04 to 9.36)	Ref.	3.53 (1.15 to 10.9)
Polydactyly	Ref.	0.92 (0.48 to 1.73)	Ref.	0.95 (0.49 to 1.85)
Syndactyly	Ref.	0.69 (0.22 to 2.14)	Ref.	0.77 (0.23 to 2.57)
Urogenital				
Hypospadias^a^	Ref.	1.56 (0.45 to 5.45)	Ref.	1.69 (0.49 to 5.78)
Gastrointestinal				
Alimentary atresia^b^	Ref.	1.80 (0.80 to 4.07)	Ref.	1.97 (0.88 to 4.39)
Esophageal atresia	Ref.	1.39 (0.40 to 4.83)	Ref.	1.61 (0.45 to 5.76)
Atresia of small intestine	Ref.	2.08 (0.59 to 7.29)	Ref.	1.80 (0.48 to 6.75)
Rectal and large intestinal atresia	Ref.	2.38 (0.77 to 7.39)	Ref.	2.69 (0.87 to 8.33)
Central nervous system				
Anencephaly	Ref.	0.45 (0.18 to 1.11)	Ref.	0.53 (0.20 to 1.45)
Spina bifida	Ref.	1.82 (0.73 to 4.52)	Ref.	1.73 (0.64 to 4.70)
Orofacial				
Cleft lip with and without cleft palate	Ref.	1.39 (0.73 to 2.64)	Ref.	1.28 (0.66 to 2.46)

*OR* odds ratio, *CI* confidence interval, *IVF* in vitro fertilization, *ICSI* intracytoplasmic sperm injection

^a^Analysis was restricted within male infants

^b^Alimentary atresia is a composite outcomes of esophageal atresia, atresia of small intestine and rectal and large intestinal atresia

^c^adjusted for maternal age, calendar year, embryo stage at transfer, and fetal sex

**Table 5:** ORs and 95% CIs of ICSI compared with IVF for major birth defects among subfertile men

Type of major birth defect	Crude OR (95% CI)		Adjusted OR (95% CI)^c^	
	IVF	ICSI	IVF	ICSI
Any major anomaly	Ref.	0.86 (0.57 to 1.31)	Ref.	0.86 (0.56 to 1.32)
Cardiovascular				
Ventricular septal defect	Ref.	0.86 (0.46 to 1.62)	Ref.	0.84 (0.44 to 1.58)
Atrial septal defect	Ref.	0.39 (0.11 to 1.32)	Ref.	0.36 (0.10 to 1.26)
Tetralogy of Fallot	Ref.	0.33 (0.05 to 2.02)	Ref.	0.35 (0.05 to 2.39)
Musculoskeletal				
Omphalocele	Ref.	0.44 (0.04 to 4.9)	Ref.	0.38 (0.05 to 2.9)
Gastroschisis	Ref.	–	Ref.	–
Diaphragmatic hernia	Ref.	0.66 (0.07 to 6.43)	Ref.	0.63 (0.06 to 6.56)
Polydactyly	Ref.	0.55 (0.11 to 2.85)	Ref.	0.55 (0.11 to 2.69)
Syndactyly	Ref.	–	Ref.	–
Urogenital				
Hypospadias^a^	Ref.	0.72 (0.21 to 2.44)	Ref.	0.67 (0.20 to 2.22)
Gastrointestinal				
Alimentary atresia^b^	Ref.	3.31 (0.43 to 25.4)	Ref.	3.05 (0.39 to 24.1)
Esophageal atresia	Ref.	–	Ref.	–
Atresia of small intestine	Ref.	–	Ref.	–
Rectal and large intestinal atresia	Ref.	1.98 (0.25 to 15.7)	Ref.	2.10 (0.24 to 18.6)
Central nervous system				
Anencephaly	Ref.	–	Ref.	–
Spina bifida	Ref.	–	Ref.	–
Orofacial				
Cleft lip with and without cleft palate	Ref.	0.81 (0.23 to 2.78)	Ref.	0.80 (0.23 to 2.77)

*OR* odds ratio, *CI* confidence interval, *IVF* in vitro fertilization, *ICSI* intracytoplasmic sperm injection

^a^Analysis was restricted within male infants

^b^Alimentary atresia is a composite outcomes of esophageal atresia, atresia of small intestine and rectal and large intestinal atresia

^c^adjusted for maternal age, calendar year, embryo stage at transfer, and fetal sex

We also calculated the ORs of oligozoospermia for major birth defects among IVF cycles for which semen parameters were available (See Additional file 1: Table S1). Oligozoospermia significantly increased the risk of any major birth defect (14/868 [1.61%] vs 277/28,090 [0.99%], adjusted OR = 1.77, 95% CI 1.08–2.90, *P* = 0.02) and of ventricular septal defects (5/868 [0.58%] vs 60/28,090 [0.21%], adjusted OR = 2.68, 95% CI 1.15–6.27, P = 0.02) compared with normal sperm concentrations.

Similarly, among ICSI cycles for which semen parameters were available, severe oligozoospermia (i.e., sperm concentrations < 5 × 10^6^/mL) was associated with a significantly increased risk of hypospadias (5/3136 [0.16%] vs 5/16,865 [0.03%], adjusted OR = 3.88, 95% CI 1.14–13.2, *P* = 0.03) compared with normal sperm concentrations, but oligozoospermia (i.e., sperm concentrations ≥5 × 10^6^/mL and < 15 × 10^6^/mL) (2/4009 [0.05%], adjusted OR = 1.63, 95% CI 0.31–8.49, *P* = 0.56) was not associated with increased risks (Additional file 2: Table S2). ICSI was not associated with any specific major birth defects compared with IVF among cycles with normal semen concentrations and motility (See Additional file 3: Table S3).

Finally, we also calculated the ORs of asthenozoospermia compared with normal sperm motility for major birth defects among ICSI and IVF cycles (See Additional file 4: Table S4 and Additional file 5: Table S5). Neither asthenozoospermia nor severe asthenozoospermia was associated with any specific major birth defects compared with normal sperm motility in either ICSI or IVF cycles.

## Discussion

This exploratory study, based on a nationally representative ART sample in Japan, showed that male subfertility was associated with greater risks of hypospadias in babies born following IVF and ICSI. Further, male subfertility was associated with significantly greater risks of atrial and ventricular septal defects among babies born after IVF. The results of this study suggest that underlying male subfertility might affect the risk of major birth defects in relation to ICSI or IVF.

Few studies to date have investigated the effects of male subfertility on the risks of major birth defects following IVF and ICSI [10]. Notably, no previous studies have reported an association between male subfertility and major birth defects following IVF, and few studies have investigated the effects of male subfertility on the risk of major birth defects following ICSI. A case-control study of 208 children born after ICSI and 221 normally conceived controls showed that ICSI using oligospermic sperm was associated with a higher overall birth-defect rate (25/121 [20.7%]) than ICSI using non-oligospermic sperm (7/87 [8.0%]) (*P* = 0.02) [15]. This previous study suggested that genitourinary birth defects were more frequent in ICSI using oligospermic sperm, although their small sample size prevented an accurate assessment of the association. Another study that investigated 2545 pregnancies conceived after ICSI using ejaculated sperm and 206 using non-ejaculated sperm [16] found no significant association between the risk of overall major birth defects and sperm concentration or the indication for ICSI, though the study did not stratify the analyses according to multiplicity and the specific type of birth defect.

A recent meta-analysis reported a significantly increased risk of overall genitourinary birth defects after ICSI compared with IVF [8]. Although the study failed to demonstrate an association between ICSI and hypospadias, this may be because the effects of paternal subfertility might have been diluted by the recent wider use of ICSI for indications other than male subfertility [17].

Male subfertility was also associated with increased risks of atrial and ventricular septal defects in IVF, but not ICSI cycles in the current study. A recent meta-analysis showed that these major birth defects were more frequent in IVF and ICSI cycles compared with spontaneous pregnancies [18]. Although they were unable to stratify ICSI and IVF in their analysis, their results suggested that parental subfertility might increase the risks of atrial and ventricular septal defects. The reason that the relationship between male subfertility and atrial and ventricular septal defect was only observed in IVF cycles was unknown. Although there is currently no relevant evidence, the effect of male subfertility could be exaggerated in IVF using spontaneous fertilization compared with ICSI, in which a single sperm is intentionally selected for artificial fertilization.

Among fertile men, ICSI was associated with a higher risk of diaphragmatic hernias compared with IVF. Although this finding was not observed in subgroup analyses of normal sperm concentrations and motility, it suggests that factors other than male subfertility, such as underlying maternal factors, may affect the incidence of this anomaly in babies born following ICSI. The registry does not contain information on maternal body mass index, smoking status, alcohol intake during pregnancy, and socioeconomic status, and it is possible that these confounding factors might have affected our results.

This study provided the first attempt to distinguish between the effects of treatment and of underlying male subfertility in relation to the risk of major birth defects in children born after IVF and ICSI; however, the study had several limitations. First, the rates of total major birth defects and specific birth defects were low compared with some other studies [4, 7], suggesting that the ascertainment of outcomes would be low. The small numbers of some birth defects led to insufficient statistical power, and the CIs for several specific major birth defects were wide. Nevertheless, the risk of hypospadias among offspring of subfertile men or cycles using oligozoospermic sperm was significantly increased following either IVF and ICSI, and was not related to the ICSI intervention itself. The prevalence of major birth defects in this study, including 279 cases of chromosomal abnormalities, was 1.51%, which was lower than the prevalence of major or minor birth defects of 2.34% among 108,087 births reported by the Japanese branch of the International Clearinghouse for Birth Defects Surveillance and Research [19, 20]. The methods of collecting outcome data might vary across ART clinics, which would introduce bias to the results. Second, male subfertility was not subdivided according to severity or type, as in other registry databases [21]. Third, although the registry included multiple deliveries from the same parents over the study period, we were not able to identify these cases, and associations within parents might thus have affected the results. Lastly, the registry lacked information on important confounders such as parity, paternal age, duration of infertility, body mass index, and proportions of teratozoospermia, which may have given rise to the possibility of residual confounding in our results. Further studies, especially systematic reviews of observational studies including the current study adjusting for important confounders, are therefore required to confirm these initial findings.

## Conclusions

In conclusion, the current exploratory analysis of data from a nationally representative ART sample in Japan suggested that male subfertility may contribute to the risks of major birth defects in relation to ICSI and IVF. Notably, male subfertility was associated with a greater risk of hypospadias as a major birth defect in children born following IVF and ICSI. However, further studies are needed to confirm the role of male subfertility in the risks of major cardiac and urogenital birth defects following IVF and ICSI, including systematic reviews and meta-analyses stratified according to the severity or type of male subfertility and adjusting for important confounders.

## Additional files

Additional file 1: **Table S1.** ORs and 95% CIs of different thresholds for sperm concentrations for major birth defects among IVF cycles for which semen parameters were available (*n* = 28,958 cycles). (DOCX 103 kb)

Additional file 2: **Table S2.** ORs and 95% CIs of different thresholds for sperm concentrations for major birth defects among ICSI cycles for which semen parameters were available (*n* = 24,010 cycles). (DOCX 106 kb)

Additional file 3: **Table S3.** ORs and 95% CIs for major birth defects among ICSI cycles with normal semen concentration and motility (*n* = 34,839 cycles). (DOCX 109 kb)

Additional file 4: **Table S4.** ORs and 95% CIs of different thresholds for sperm motility for major birth defects among IVF cycles for which semen parameters were available (n = 28,958 cycles). (DOCX 122 kb)

Additional file 5: **Table S5.** ORs and 95% CIs of different thresholds for sperm motility for major birth defects among ICSI cycles for which semen parameters were available (n = 24,010 cycles). (DOCX 131 kb)

## Abbreviations

ART: assisted reproductive technology
CDC: US Centers for Disease Control and Prevention
CI: confidence interval
ICSI: intracytoplasmic sperm injection
IVF: in vitro fertilization
JSOG: the Japan Society of Obstetrics and Gynecology
OR: odds ratio
