# Supplementary material for: Male subfertility and the risk of major birth defects in children born after in vitro fertilization and intracytoplasmic sperm injection: a retrospective cohort study
Source: BMC Pregnancy Childbirth. 2019 Jun 3;19:192. doi: 10.1186/s12884-019-2322-7 (PMC6547560; doi:10.1186/s12884-019-2322-7)
Supplement: Supplementary file 1 — Table S1. ORs and 95% CIs of different thresholds for sperm concentrations for major birth defects among IVF cycles for which semen parameters were available (n = 28,958 cycles). (DOCX 103 kb) [file 12884_2019_2322_MOESM1_ESM.docx]

| **Additional table 1.** ORs and 95% CIs of different thresholds for sperm concentrations for major birth defects within IVF among cycles for which semen parameters were available (n=28,958 cycles). | | |
| --- | --- | --- |
| Type of major birth defect | IVF | |
|  | Normal sperm concentration (n=28,090) | Oligozoospermia^a^ (n=868) |
| **Any major anomaly, n(%)** | 277 (0.99) | 14 (1.61) |
| Crude OR (95% CI) | Ref. | 1.65 (1.02 to 2.66) |
| Adjusted OR (95% CI)^d^ | Ref. | 1.77 (1.08 to 2.90) |
|  |  |  |
| **Cardiovascular** |  |  |
| Ventricular septal defect, n(%) | 60 (0.21) | 5 (0.58) |
| Crude OR (95% CI) | Ref. | 2.71 (1.18 to 6.21) |
| Adjusted OR (95% CI)^d^ | Ref. | 2.68 (1.15 to 6.27) |
| Atrial septal defect, n(%) | 12 (0.04) | 1 (0.12) |
| Crude OR (95% CI) | Ref. | 2.70 (0.83 to 8.81) |
| Adjusted OR (95% CI)^d^ | Ref. | 2.50 (0.74 to 8.46) |
| Tetralogy of Fallot, n(%) | 11 (0.04) | 1 (0.12) |
| Crude OR (95% CI) | Ref. | 2.94 (0.39 to 22.4) |
| Adjusted OR (95% CI)^d^ | Ref. | 3.09 (0.38 to 25.0) |
| **Musculoskeletal** |  |  |
| Omphalocele, n(%) | 2 (0.01) | 0 (0) |
| Crude OR (95% CI) | Ref. | – |
| Adjusted OR (95% CI)^d^ | Ref. | – |
| Gastroschisis, n(%) | 1 (0) | 1 (0.12) |
| Crude OR (95% CI) | Ref. | – |
| Adjusted OR (95% CI)^d^ | Ref. | – |
| Diaphragmatic hernia, n(%) | 5 (0.02) | 0 (0) |
| Crude OR (95% CI) | Ref. | – |
| Adjusted OR (95% CI)^d^ | Ref. | – |
| Polydactyly, n(%) | 23 (0.08) | 2 (0.23) |
| Crude OR (95% CI) | Ref. | 2.82 (0.39 to 20.2) |
| Adjusted OR (95% CI)^d^ | Ref. | 3.06 (0.43 to 21.9) |
| Syndactyly, n(%) | 9 (0.03) | 0 (0) |
| Crude OR (95% CI) | Ref. | – |
| Adjusted OR (95% CI)^d^ | Ref. | – |
| **Urogenital** |  |  |
| Hypospadias, n(%)^b^ | 6 (0.02) | 0 (0) |
| Crude OR (95% CI) | Ref. | – |
| Adjusted OR (95% CI)^d^ | Ref. | – |
| **Gastrointestinal** |  |  |
| Alimentary atresia, n(%)^c^ | 15 (0.05) | 1 (0.12) |
| Crude OR (95% CI) | Ref. | 2.16 (0.27 to 17.0) |
| Adjusted OR (95% CI)^d^ | Ref. | 2.23 (0.28 to 17.7) |
| Esophageal atresia, n(%) | 6 (0.02) | 0 (0) |
| Crude OR (95% CI) | Ref. | – |
| Adjusted OR (95% CI)^d^ | Ref. | – |
| Atresia of small intestine, n(%) | 2 (0.01) | 0 (0) |
| Crude OR (95% CI) | Ref. | – |
| Adjusted OR (95% CI)^d^ | Ref. | – |
| Rectal and large intestinal atresia, n(%) | 7 (0.02) | 1 (0.12) |
| Crude OR (95% CI) | Ref. | 4.63 (0.54 to 39.4) |
| Adjusted OR (95% CI)^d^ | Ref. | – |
| **Central nervous system** |  |  |
| Anencephaly, n(%) | 18 (0.06) | 2 (0.23) |
| Crude OR (95% CI) | Ref. | 3.60 (1.15 to 11.3) |
| Adjusted OR (95% CI)^d^ | Ref. | – |
| Spina bifida, n(%) | 8 (0.03) | 0 (0) |
| Crude OR (95% CI) | Ref. | – |
| Adjusted OR (95% CI)^d^ | Ref. | – |
| **Orofacial** |  |  |
| Cleft lip with and without cleft palate, n(%) | 19 (0.07) | 0 (0) |
| Crude OR (95% CI) | Ref. | – |
| Adjusted OR (95% CI)^d^ | Ref. | – |
| OR=odds ratio; CI=confidence interval; IVF=*in vitro* fertilization; ICSI=intracytoplasmic sperm injection. | | |
| ^a^ Oligozoospermia was defined as sperm concentrations <15×10^6^spermatozoa/mL. | | |
| ^b^ Analysis was restricted within male infants. | | |
| ^c^ Alimentary atresia is a composite outcomes of esophageal atresia, atresia of small intestine and rectal and large intestinal atresia. | | |
| ^d^ adjusted for maternal age, calendar year, embryo stage at transfer, and fetal sex. | | |
