# Supplementary material for: Male subfertility and the risk of major birth defects in children born after in vitro fertilization and intracytoplasmic sperm injection: a retrospective cohort study
Source: BMC Pregnancy Childbirth. 2019 Jun 3;19:192. doi: 10.1186/s12884-019-2322-7 (PMC6547560; doi:10.1186/s12884-019-2322-7)
Supplement: Supplementary file 2 — Table S2. ORs and 95% CIs of different thresholds for sperm concentrations for major birth defects among ICSI cycles for which semen parameters were available (n = 24,010 cycles). (DOCX 106 kb) [file 12884_2019_2322_MOESM2_ESM.docx]

| **Additional table 2.** ORs and 95% CIs of different thresholds for sperm concentrations for major birth defects within ICSI among cycles for which semen parameters were available (n=24,010 cycles). | | | |
| --- | --- | --- | --- |
| Type of major birth defect | ICSI | | |
|  | Normal sperm concentration (n=16,865) | Oligozoospermia^a^ (n=4009) | Severe oligozoospermia^a^ (n=3136) |
| **Any major anomaly, n(%)** | 189 (1.12) | 50 (1.25) | 37 (1.18) |
| Crude OR (95% CI) | Ref. | 1.11 (0.82 to 1.52) | 1.05 (0.76 to 1.45) |
| Adjusted OR (95% CI)^d^ | Ref. | 1.10 (0.75 to 1.43) | 1.04 (0.75 to 1.43) |
|  |  |  |  |
| **Cardiovascular** |  |  |  |
| Ventricular septal defect, n(%) | 53 (0.31) | 12 (0.30) | 7 (0.22) |
| Crude OR (95% CI) | Ref. | 0.95 (0.54 to 1.68) | 0.71 (0.36 to 1.38) |
| Adjusted OR (95% CI)^d^ | Ref. | 0.97 (0.54 to 1.72) | 0.74 (0.38 to 1.46) |
| Atrial septal defect, n(%) | 10 (0.06) | 2 (0.05) | 3 (0.10) |
| Crude OR (95% CI) | Ref. | 0.84 (0.24 to 2.96) | 1.61 (0.46 to 5.64) |
| Adjusted OR (95% CI)^d^ | Ref. | 0.90 (0.25 to 3.21) | 1.70 (0.49 to 5.97) |
| Tetralogy of Fallot, n(%) | 2 (0.01) | 2 (0.05) | 1 (0.03) |
| Crude OR (95% CI) | Ref. | 4.39 (0.67 to 28.6) | 3.08 (0.33 to 28.5) |
| Adjusted OR (95% CI)^d^ | Ref. | 4.48 (0.68 to 29.7) | 3.12 (0.35 to 28.1) |
| **Musculoskeletal** |  |  |  |
| Omphalocele, n(%) | 2 (0.01) | 0 (0.0) | 1 (0.03) |
| Crude OR (95% CI) | Ref. | – | 2.69 (0.24 to 30.4) |
| Adjusted OR (95% CI)^d^ | Ref. | – | 2.82 (0.24 to 32.8) |
| Gastroschisis, n(%) | 0 (0) | 0 (0) | 0 (0) |
| Crude OR (95% CI) | Ref. | – | – |
| Adjusted OR (95% CI)^d^ | Ref. | – | – |
| Diaphragmatic hernia, n(%) | 5 (0.03) | 3 (0.07) | 1 (0.03) |
| Crude OR (95% CI) | Ref. | 2.53 (0.61 to 10.5) | 1.08 (0.12 to 10.5) |
| Adjusted OR (95% CI)^d^ | Ref. | 2.66 (0.59 to 11.9) | 1.17 (0.13 to 10.3) |
| Polydactyly, n(%) | 12 (0.07) | 2 (0.05) | 0 (0) |
| Crude OR (95% CI) | Ref. | 0.70 (0.18 to 2.71) | – |
| Adjusted OR (95% CI)^d^ | Ref. | 0.66 (0.17 to 2.50) | – |
| Syndactyly, n(%) | 4 (0.02) | 2 (0.05) | 0 (0) |
| Crude OR (95% CI) | Ref. | 2.10 (0.41 to 10.88) | – |
| Adjusted OR (95% CI)^d^ | Ref. | 2.20 (0.45 to 10.8) | – |
| **Urogenital** |  |  |  |
| Hypospadias, n(%)^b^ | 5 (0.03) | 2 (0.05) | 5 (0.16) |
| Crude OR (95% CI) | Ref. | 1.69 (0.33 to 8.67) | 4.24 (1.13 to 15.9) |
| Adjusted OR (95% CI)^d^ | Ref. | 1.63 (0.31 to 8.49) | 3.88 (1.14 to 13.2) |
| **Gastrointestinal** |  |  |  |
| Alimentary atresia, n(%)^c^ | 18 (0.11) | 2 (0.05) | 3 (0.10) |
| Crude OR (95% CI) | Ref. | 0.47 (0.10 to 2.09) | 0.90 (0.27 to 2.94) |
| Adjusted OR (95% CI)^d^ | Ref. | 0.44 (0.10 to 1.95) | 0.76 (0.24 to 2.48) |
| Esophageal atresia, n(%) | 5 (0.03) | 1 (0.02) | 0 (0) |
| Crude OR (95% CI) | Ref. | 0.84 (0.099 to 7.17) | – |
| Adjusted OR (95% CI)^d^ | Ref. | 0.76 (0.098 to 5.85) | – |
| Atresia of small intestine, n(%) | 4 (0.02) | 0 (0) | 0 (0) |
| Crude OR (95% CI) | Ref. | – | – |
| Adjusted OR (95% CI)^d^ | Ref. | – | – |
| Rectal and large intestinal atresia, n(%) | 10 (0.06) | 1 (0.02) | 3 (0.10) |
| Crude OR (95% CI) | Ref. | 0.42 (0.053 to 3.33) | 1.61 (0.48 to 5.42) |
| Adjusted OR (95% CI)^d^ | Ref. | 0.41 (0.05 to 3.38) | 1.41 (0.42 to 4.71) |
| **Central nervous system** |  |  |  |
| Anencephaly, n(%) | 5 (0.03) | 4 (0.10) | 1 (0.03) |
| Crude OR (95% CI) | Ref. | 3.37 (1.06 to 10.7) | 1.08 (0.13 to 9.19) |
| Adjusted OR (95% CI)^d^ | Ref. | 2.94 (0.89 to 9.66) | 0.67 (0.07 to 6.38) |
| Spina bifida, n(%) | 8 (0.05 ) | 0 (0) | 1 (0.03) |
| Crude OR (95% CI) | Ref. | – | 0.67 (0.084 to 5.38) |
| Adjusted OR (95% CI)^d^ | Ref. | – | 0.70 (0.08 to 5.71) |
| **Orofacial** |  |  |  |
| Cleft lip with and without cleft palate, n(%) | 17 (0.10) | 3 (0.07) | 2 (0.06) |
| Crude OR (95% CI) | Ref. | 0.74 (0.21 to 2.61) | 0.63 (0.14 to 2.86) |
| Adjusted OR (95% CI)^d^ | Ref. | 0.77 (0.22 to 2.68) | 0.70 (0.14 to 3.26) |
| OR=odds ratio; CI=confidence interval; IVF=*in vitro* fertilization; ICSI=intracytoplasmic sperm injection. | | | |
| ^a^ Oligozoospermia was defined as sperm concentrations <15×10^6^spermatozoa/mL and severe oligozoospermia was defined as sperm concentrations <5×10^6^spermatozoa/mL. | | | |
| ^b^ Analysis was restricted within male infants. | | | |
| ^c^ Alimentary atresia is a composite outcomes of esophageal atresia, atresia of small intestine and rectal and large intestinal atresia. | | | |
| ^d^ adjusted for maternal age, calendar year, embryo stage at transfer, and fetal sex. | | |  |
