# Supplementary material for: Male subfertility and the risk of major birth defects in children born after in vitro fertilization and intracytoplasmic sperm injection: a retrospective cohort study
Source: BMC Pregnancy Childbirth. 2019 Jun 3;19:192. doi: 10.1186/s12884-019-2322-7 (PMC6547560; doi:10.1186/s12884-019-2322-7)
Supplement: Supplementary file 3 — Table S3. ORs and 95% CIs for major birth defects among ICSI cycles with normal semen concentration and motility (n = 34,839 cycles). (DOCX 109 kb) [file 12884_2019_2322_MOESM3_ESM.docx]

| **Additional table 3.** ORs and 95% CIs of ICSI for major birth defects among cycles for which semen concentration and motility were normal (n=34,839 cycles). | | |
| --- | --- | --- |
| Type of major birth defect | IVF (n=24,319) | ICSI (n=10,520) |
| **Any major anomaly, n(%)** | 244 (1.00) | 120 (1.14) |
| Crude OR (95% CI) | Ref. | 1.14 (0.91 to 1.42) |
| Adjusted OR (95% CI)^d^ | Ref. | !.17 (0.93 to 1.46) |
|  |  |  |
| **Cardiovascular** |  |  |
| Ventricular septal defect, n(%) | 52 (0.21) | 30 (0.29) |
| Crude OR (95% CI) | Ref. | 1.33 (0.86 to 2.08) |
| Adjusted OR (95% CI)^d^ | Ref. | 1.30 (0.83 to 2.03) |
| Atrial septal defect, n(%) | 12 (0.05) | 6 (0.06) |
| Crude OR (95% CI) | Ref. | 1.16 (0.52 to 2.57) |
| Adjusted OR (95% CI)^d^ | Ref. | 1.19 (0.51 to 2.79) |
| Tetralogy of Fallot, n(%) | 8 (0.03) | 1 (0.01) |
| Crude OR (95% CI) | Ref. | 0.29 (0.04 to 2.33) |
| Adjusted OR (95% CI)^d^ | Ref. | 0.26 (0.033 to 2.09) |
| **Musculoskeletal** |  |  |
| Omphalocele, n(%) | 1 (0.004) | 1 (0.01) |
| Crude OR (95% CI) | Ref. | 2.31 (0.14 to 37.2) |
| Adjusted OR (95% CI)^d^ | Ref. | – |
| Gastroschisis, n(%) | 1 (0.004) | 0 (0) |
| Crude OR (95% CI) | Ref. | – |
| Adjusted OR (95% CI)^d^ | Ref. | – |
| Diaphragmatic hernia, n(%) | 5 (0.02) | 3 (0.03) |
| Crude OR (95% CI) | Ref. | 1.39 (0.29 to 6.57) |
| Adjusted OR (95% CI)^d^ | Ref. | 1.47 (0.29 to 7.34) |
| Polydactyly, n(%) | 21 (0.09) | 8 (0.08) |
| Crude OR (95% CI) | Ref. | 0.88 (0.44 to 1.78) |
| Adjusted OR (95% CI)^d^ | Ref. | 0.89 (0.44 to 1.82) |
| Syndactyly, n(%) | 8 (0.03) | 3 (0.03) |
| Crude OR (95% CI) | Ref. | 0.87 (0.24 to 3.13) |
| Adjusted OR (95% CI)^d^ | Ref. | 0.93 (0.25 to 3.44) |
| **Urogenital** |  |  |
| Hypospadias, n(%)^b^ | 5 (0.02) | 5 (0.05) |
| Crude OR (95% CI) | Ref. | 2.31 (0.87 to 6.18) |
| Adjusted OR (95% CI)^d^ | Ref. | 2.38 (0.87 to 6.53) |
| **Gastrointestinal** |  |  |
| Alimentary atresia, n(%)^c^ | 15 (0.06) | 10 (0.10) |
| Crude OR (95% CI) | Ref. | 1.54 (0.70 to 3.39) |
| Adjusted OR (95% CI)^d^ | Ref. | 1.65 (0.75 to 3.65) |
| Esophageal atresia, n(%) | 6 (0.02) | 2 (0.02) |
| Crude OR (95% CI) | Ref. | 0.77 (0.16 to 3.82) |
| Adjusted OR (95% CI)^d^ | Ref. | 0.86 (0.16 to 4.52) |
| Atresia of small intestine, n(%) | 2 (0.01) | 2 (0.02) |
| Crude OR (95% CI) | Ref. | 2.31 (0.56 to 9.59) |
| Adjusted OR (95% CI)^d^ | Ref. | 1.94 (0.49 to 7.76) |
| Rectal and large intestinal atresia, n(%) | 7 (0.03) | 6 (0.06) |
| Crude OR (95% CI) | Ref. | 1.98 (0.59 to 6.67) |
| Adjusted OR (95% CI)^d^ | Ref. | 2.23 (0.66 to 7.49) |
| **Central nervous system** |  |  |
| Anencephaly, n(%) | 16 (0.07) | 2 (0.02) |
| Crude OR (95% CI) | Ref. | 0.29 (0.06 to 1.31) |
| Adjusted OR (95% CI)^d^ | Ref. | 0.36 (0.08 to 1.62) |
| Spina bifida, n(%) | 7 (0.03) | 7 (0.07) |
| Crude OR (95% CI) | Ref. | 2.31 (0.97 to 5.49) |
| Adjusted OR (95% CI)^d^ | Ref. | 2.41 (0.95 to 6.15) |
| **Orofacial** |  |  |
| Cleft lip with and without cleft palate, n(%) | 16 (0.07) | 10 (0.10) |
| Crude OR (95% CI) | Ref. | 1.45 (0.65 to 3.23) |
| Adjusted OR (95% CI)^d^ | Ref. | 1.42 (0.65 to 3.12) |
| OR=odds ratio; CI=confidence interval; IVF=*in vitro* fertilization; ICSI=intracytoplasmic sperm injection; VSD=ventricular septal defect; ASD=atrial septal defect. | | |
| ^a^ analysis was restricted within male infants. |  |  |
| ^b^ Alimentary atresia is a composite outcomes of esophageal atresia, atresia of small intestine and rectal and large intestinal atresia. | | |
| ^d^ adjusted for maternal age, calendar year, embryo stage at transfer, and fetal sex. | | |
