# Supplementary material for: Male subfertility and the risk of major birth defects in children born after in vitro fertilization and intracytoplasmic sperm injection: a retrospective cohort study
Source: BMC Pregnancy Childbirth. 2019 Jun 3;19:192. doi: 10.1186/s12884-019-2322-7 (PMC6547560; doi:10.1186/s12884-019-2322-7)
Supplement: Supplementary file 4 — Table S4. ORs and 95% CIs of different thresholds for sperm motility for major birth defects among IVF cycles for which semen parameters were available (n = 28,958 cycles). (DOCX 122 kb) [file 12884_2019_2322_MOESM4_ESM.docx]

| **Additional table 4.** ORs and 95% CIs of different thresholds for sperm motility for major birth defects in IVF among cycles for which semen parameters were available (n=28,958 cycles). | | | |
| --- | --- | --- | --- |
| Type of major birth defect | IVF | | |
|  | Normal sperm motility (n=24,935) | Asthenozoospermia^a^ (n=3078) | Severe asthenozoospermia^a^ (n=945) |
| **Any major anomaly, n(%)** | 253 (1.01) | 28 (0.91) | 10 (1.06) |
| Crude OR (95% CI) | Ref. | 0.90 (0.59 to 1.35) | 1.04 (0.58 to 1.87) |
| Adjusted OR (95% CI)^d^ | Ref. | 0.87 (0.58 to 1.32) | 1.11 (0.63 to 1.97) |
|  |  |  |  |
| **Cardiovascular** |  |  |  |
| Ventricular septal defect, n(%) | 54 (0.22) | 7 (0.23) | 4 (0.42) |
| Crude OR (95% CI) | Ref. | 1.05 (0.44 to 2.49) | 1.96 (0.71 to 5.43) |
| Adjusted OR (95% CI)^d^ | Ref. | 1.02 (0.43 to 2.42) | 1.83 (0.66 to 5.11) |
| Atrial septal defect, n(%) | 13 (0.05) | 0 (0) | 0 (0) |
| Crude OR (95% CI) | Ref. | – | – |
| Adjusted OR (95% CI)^d^ | Ref. | – | – |
| Tetralogy of Fallot, n(%) | 9 (0.04) | 2 (0.06) | 1 (0.11) |
| Crude OR (95% CI) | Ref. | 1.80 (0.39 to 8.30) | 2.93 (0.38 to 22.8) |
| Adjusted OR (95% CI)^d^ | Ref. | 1.79 (0.39 to 8.17) | 2.83 (0.37 to 21.7) |
| **Musculoskeletal** |  |  |  |
| Omphalocele, n(%) | 1 (0.00) | 1 (0.03) | 0 (0) |
| Crude OR (95% CI) | Ref. | 8.10 (0.49 to 133.2) | – |
| Adjusted OR (95% CI)^d^ | Ref. | – | – |
| Gastroschisis, n(%) | 1 (0.00) | 1 (0.03) | 0 (0) |
| Crude OR (95% CI) | Ref. | 8.10 (0.51 to 127.6) | – |
| Adjusted OR (95% CI)^d^ | Ref. | – | – |
| Diaphragmatic hernia, n(%) | 5 (0.02) | 0 (0) | 0 (0) |
| Crude OR (95% CI) | Ref. | – | – |
| Adjusted OR (95% CI)^d^ | Ref. | – | – |
| Polydactyly, n(%) | 23 (0.09) | 1 (0.03) | 1 (0.11) |
| Crude OR (95% CI) | Ref. | 0.35 (0.052 to 2.40) | 1.15 (0.15 to 8.54) |
| Adjusted OR (95% CI)^d^ | Ref. | 0.36 (0.052 to 2.43) | 1.18 (0.16 to 8.74) |
| Syndactyly, n(%) | 8 (0.03) | 0 (0) | 1 (0.11) |
| Crude OR (95% CI) | Ref. | – | 3.30 (0.42 to 26.2) |
| Adjusted OR (95% CI)^d^ | Ref. | – | 3.08 (0.39 to 24.2) |
| **Urogenital** |  |  |  |
| Hypospadias, n(%)^b^ | 5 (0.02) | 1 (0.03) | 0 (0) |
| Crude OR (95% CI) | Ref. | 1.64 (0.19 to 14.0) | – |
| Adjusted OR (95% CI)^d^ | Ref. | 1.51(0.17 to 13.1) | – |
| **Gastrointestinal** |  |  |  |
| Alimentary atresia, n(%)^c^ | 15 (0.06) | 1 (0.03) | 0 (0) |
| Crude OR (95% CI) | Ref. | 0.54 (0.07 to 4.19) | – |
| Adjusted OR (95% CI)^d^ | Ref. | 0.54 (0.07 to 4.12) | – |
| Esophageal atresia, n(%) | 6 (0.02) | 0 (0) | 0 (0) |
| Crude OR (95% CI) | Ref. | – | – |
| Adjusted OR (95% CI)^d^ | Ref. | – | – |
| Atresia of small intestine, n(%) | 2 (0.01) | 0 (0) | 0 (0) |
| Crude OR (95% CI) | Ref. | – | – |
| Adjusted OR (95% CI)^d^ | Ref. | – | – |
| Rectal and large intestinal atresia, n(%) | 7 (0.03) | 1 (0.03) | 0 (0) |
| Crude OR (95% CI) | Ref. | 1.16 (0.14 to 9.87) | – |
| Adjusted OR (95% CI)^d^ | Ref. | 1.23 (0.15 to 10.2) | – |
| **Central nervous system** |  |  |  |
| Anencephaly, n(%) | 18 (0.07) | 0 (0) | 2 (0.21) |
| Crude OR (95% CI) | Ref. | – | 2.94 (0.67 to 12.8) |
| Adjusted OR (95% CI)^d^ | Ref. | – | – |
| Spina bifida, n(%) | 7 (0.03) | 1 (0.03) | 0 (0) |
| Crude OR (95% CI) | Ref. | 1.16 (0.13 to 10.1) | – |
| Adjusted OR (95% CI)^d^ | Ref. | 0.98 (0.14 to 6.96) | – |
| **Orofacial** |  |  |  |
| Cleft lip with and without cleft palate, n(%) | 16 (0.06) | 3 (0.10) | 0 (0) |
| Crude OR (95% CI) | Ref. | 1.52 (0.44 to 5.26) | – |
| Adjusted OR (95% CI)^d^ | Ref. | 1.49 (0.43 to 5.11) | – |
| OR=odds ratio; CI=confidence interval; IVF=*in vitro* fertilization; ICSI=intracytoplasmic sperm injection; VSD=ventricular septal defect; ASD=atrial septal defect. | | | |
| ^a^ Asthenozoospermia was defined as total sperm motility <40% and severe asthenozoospermia was defined as total sperm motility <25% | | | |
| ^b^ analysis was restricted within male infants. | |  |  |
| ^c^ Alimentary atresia is a composite outcomes of esophageal atresia, atresia of small intestine and rectal and large intestinal atresia. | | | |
| ^d^ adjusted for maternal age, calendar year, embryo stage at transfer, and fetal sex. | | |  |
