# Supplementary material for: Male subfertility and the risk of major birth defects in children born after in vitro fertilization and intracytoplasmic sperm injection: a retrospective cohort study
Source: BMC Pregnancy Childbirth. 2019 Jun 3;19:192. doi: 10.1186/s12884-019-2322-7 (PMC6547560; doi:10.1186/s12884-019-2322-7)
Supplement: Supplementary file 5 — Table S5. ORs and 95% CIs of different thresholds for sperm motility for major birth defects among ICSI cycles for which semen parameters were available (n = 24,010 cycles). (DOCX 131 kb) [file 12884_2019_2322_MOESM5_ESM.docx]

| **Additional table 5.** ORs and 95% CIs of different thresholds for sperm motility for major birth defects in ICSI among cycles for which semen parameters were available (n=24,010 cycles). | | | |
| --- | --- | --- | --- |
| Type of major birth defect | ICSI | | |
|  | Normal sperm motility (n=13,053) | Asthenozoospermia^a^ (n=4954) | Severe asthenozoospermia^a^ (n=6003) |
| **Any major anomaly, n(%)** | 149 (1.14) | 48 (0.97) | 79 (1.32) |
| Crude OR (95% CI) | Ref. | 0.85 (0.61 to 1.18) | 1.15 (0.88 to 1.52) |
| Adjusted OR (95% CI)^d^ | Ref. | 0.84 (0.61 to 1.17) | 1.11 (0.85 to 1.45) |
|  |  |  |  |
| **Cardiovascular** |  |  |  |
| Ventricular septal defect, n(%) | 34 (0.26) | 16 (0.32) | 22 (0.37) |
| Crude OR (95% CI) | Ref. | 1.24 (0.59 to 2.60) | 1.41 (0.83 to 2.39) |
| Adjusted OR (95% CI)^d^ | Ref. | 1.25 (0.61 to 2.59) | 1.44 (0.85 to 2.43) |
| Atrial septal defect, n(%) | 7 (0.05) | 3 (0.06) | 5 (0.08) |
| Crude OR (95% CI) | Ref. | 1.13 (0.32 to 4.02) | 1.55 (0.40 to 6.05) |
| Adjusted OR (95% CI)^d^ | Ref. | 1.19 (0.33 to 4.31) | 1.57 (0.41 to 5.98) |
| Tetralogy of Fallot, n(%) | 1 (0.01) | 3 (0.06) | 1 (0.02) |
| Crude OR (95% CI) | Ref. | 7.91 (0.82 to 76.2) | 2.17 (0.14 to 34.8) |
| Adjusted OR (95% CI)^d^ | Ref. | 8.13 (0.86 to 77.0) | 2.29 (0.15 to 35.0) |
| **Musculoskeletal** |  |  |  |
| Omphalocele, n(%) | 1 (0.01) | 0 (0.0) | 2 (0.03) |
| Crude OR (95% CI) | Ref. | – | 4.35 (0.40 to 47.6) |
| Adjusted OR (95% CI)^d^ | Ref. | – | – |
| Gastroschisis, n(%) | 0 (0) | 0 (0) | 0 (0) |
| Crude OR (95% CI) | Ref. | – | – |
| Adjusted OR (95% CI)^d^ | Ref. | – | – |
| Diaphragmatic hernia, n(%) | 4 (0.03) | 2 (0.04) | 3 (0.05) |
| Crude OR (95% CI) | Ref. | 1.32 (0.33 to 5.30) | 1.63 (0.37 to 7.15) |
| Adjusted OR (95% CI)^d^ | Ref. | 1.35 (0.34 to 5.25) | 1.66 (0.37 to 7.52) |
| Polydactyly, n(%) | 8 (0.06) | 2 (0.04) | 4 (0.07) |
| Crude OR (95% CI) | Ref. | 0.66 (0.14 to 3.10) | 1.09 (0.36 to 3.26) |
| Adjusted OR (95% CI)^d^ | Ref. | 0.66 (0.14 to 3.12) | 1.05 (0.36 to 3.07) |
| Syndactyly, n(%) | 3 (0.02) | 1 (0.02) | 2 (0.03) |
| Crude OR (95% CI) | Ref. | 0.88 (0.09 to 8.22) | 1.45 (0.24 to 8.71) |
| Adjusted OR (95% CI)^d^ | Ref. | 0.93 (0.099 to 8.67) | 1.49 (0.25 to 9.04) |
| **Urogenital** |  |  |  |
| Hypospadias, n(%)^b^ | 10 (0.08) | 0 (0) | 2 (0.03) |
| Crude OR (95% CI) | Ref. | – | 0.48 (0.12 to 1.86) |
| Adjusted OR (95% CI)^d^ | Ref. | – | 0.46 (0.12 to 1.72) |
| **Gastrointestinal** |  |  |  |
| Alimentary atresia, n(%)^c^ | 12 (0.09) | 3 (0.06) | 8 (0.13) |
| Crude OR (95% CI) | Ref. | 0.66 (0.13 to 3.22) | 1.45 (0.64 to 3.26) |
| Adjusted OR (95% CI)^d^ | Ref. | 0.66 (0.14 to 3.12) | 1.38 (0.61 to 3.15) |
| Esophageal atresia, n(%) | 3 (0.02) | 1 (0.02) | 2 (0.03) |
| Crude OR (95% CI) | Ref. | 0.88 (0.09 to 8.50) | 1.45 (0.23 to 9.01) |
| Adjusted OR (95% CI)^d^ | Ref. | 0.90 (0.09 to 9.16) | 1.38 (0.23 to 8.32) |
| Atresia of small intestine, n(%) | 2 (0.02) | 0 (0) | 2 (0.03) |
| Crude OR (95% CI) | Ref. | – | 2.17 (0.53 to 8.97) |
| Adjusted OR (95% CI)^d^ | Ref. | – | 2.31 (0.59 to 9.10) |
| Rectal and large intestinal atresia, n(%) | 7 (0.05) | 3 (0.06) | 4 (0.07) |
| Crude OR (95% CI) | Ref. | 1.13 (0.21 to 6.05) | 1.24 (0.34 to 4.51) |
| Adjusted OR (95% CI)^d^ | Ref. | 1.11 (0.23 to 5.42) | 1.14 (0.31 to 4.22) |
| **Central nervous system** |  |  |  |
| Anencephaly, n(%) | 4 (0.03) | 3 (0.06) | 3 (0.05) |
| Crude OR (95% CI) | Ref. | 1.98 (0.56 to 7.03) | 1.63 (0.37 to 7.24) |
| Adjusted OR (95% CI)^d^ | Ref. | 2.18 (0.68 to 6.99) | 1.34 (0.25 to 7.31) |
| Spina bifida, n(%) | 8 (0.06) | 0 (0) | 1 (0.02) |
| Crude OR (95% CI) | Ref. | – | 0.27 (0.03 to 2.20) |
| Adjusted OR (95% CI)^d^ | Ref. | – | 0.25 (0.03 to 2.12) |
| **Orofacial** |  |  |  |
| Cleft lip with and without cleft palate, n(%) | 13 (0.10) | 1 (0.02) | 8 (0.13) |
| Crude OR (95% CI) | Ref. | 0.20 (0.02 to 1.71) | 1.34 (0.47 to 3.77) |
| Adjusted OR (95% CI)^d^ | Ref. | 0.20 (0.02 to 1.68) | 1.32 (0.47 to 3.71) |
| OR=odds ratio; CI=confidence interval; IVF=*in vitro* fertilization; ICSI=intracytoplasmic sperm injection; VSD=ventricular septal defect; ASD=atrial septal defect. | | | |
| ^a^ Asthenozoospermia was defined as total sperm motility <40% and severe asthenozoospermia was defined as total sperm motility <25% | | | |
| ^b^ analysis was restricted within male infants. | |  |  |
| ^c^ Alimentary atresia is a composite outcomes of esophageal atresia, atresia of small intestine and rectal and large intestinal atresia. | | | |
| ^d^ adjusted for maternal age, calendar year, embryo stage at transfer, and fetal sex. | | |  |
